# Fish domestication in aquaculture: 10 unanswered questions

**DOI:** 10.1093/af/vfab012

**Published:** 2021-06-19

**Authors:** Fabrice Teletchea

**Affiliations:** Unité de Recherche Animal and Fonctionnalités des Produits Animaux, Institut National de Recherche pour l’Agriculture, l’Alimentation et l’Environnement, Université de Lorraine, 54505 Vandœuvre-lès-Nancy, France

**Keywords:** diversification, fish domestication, selective breeding programs, sustainability, global issues

**Implications**

- Aquaculture is the fastest-growing food-production sector in the world.
- The number of farmed and domesticated fish species has increased tremendously in the past decades, even though the 20 most-produced species accounted for >80% of total fish aquaculture production.
- This article discusses 10 partially unanswered questions related to fish domestication that could help enhancing the sustainable development of aquaculture.
- Domestication is a powerful tool to continue improving the production of already domesticated species and farm new species, particularly those native, which could all be better adapted to cope with economic, social, and environmental issues.

## Introduction

The first trials of farming fish species for human consumption might date back to 8,000 yr ago, with the managed aquaculture of common carp (*Cyprinus carpio*) in Henan Province, China (Nakajima et al., 2019). Evidence of farming was also found on Egyptian tombs from about 3,500 yr, with some kind of control over the reproduction of Nile tilapia (*Oreochromis niloticus*) in irrigation ponds (Teletchea, 2019a). In Europe, the farming of common carp in ponds was already developed during the Middle Ages. The Italian “Vallicoltura” (extensive farming of various marine species in coastal lagoons and large open waterbodies) dates back to the 15th century. The French trout culture started around the second half of the nineteenth century (Teletchea, 2019a). In North America, aquaculture started about 100 yr ago. In Africa, aquaculture dates back to the 1940s. In Australia, New Zealand, and diverse Pacific Island states, the development of aquaculture is even more recent. In conclusion, the rearing of fish is very old (Gjedrem et al., 2012), particularly in Asia (De Silva et al., 2009), even though this is not before the early 1980s that aquaculture truly boomed, becoming the fastest-growing food-production sector globally (Teletchea, 2016a; FAO, 2019). In only four decades, aquaculture production has surpassed capture fisheries, and today more than half of the fish destined to human consumption are farmed globally (Teletchea 2016a; FAO, 2019; Houston et al., 2020). The exponential growth of aquaculture has relied partly on the domestication of an increasing number of fish species (FAO, 2019; Teletchea, 2019b). The aim of the present article is to discuss briefly 10 partly unanswered questions linked to fish domestication, which could be taken into account to promote a more sustainable global aquaculture production.

## Question 1: What Is Fish Domestication?

There is no scientific reason to consider fish domestication differently from any other animal domestication (Balon, 2004; Bilio, 2008; Hedgecock, 2012; Lorenzen et al., 2012; Teletchea, 2015a; Saraiva et al., 2019). Therefore, fish domestication could be defined as a dynamic and endless process, which starts as soon as individuals are transferred from wild to captive conditions (Teletchea, 2015a). Five genetic processes are involved in the evolution of fish during domestication: two uncontrolled (inbreeding, genetic drift), two partially controlled (natural selection in captivity, relaxation of natural selection in captivity), and one controlled (active selection) (Teletchea, 2015a). In addition, the diverse molecular mechanisms involved in ‘nongenetic’ modes of inheritance can alter aspects of genome activity and affect progeny gene expression (Adrian-Kalchhauser et al., 2020). Summing up, domestication allows adapting continuously a batch of fish to both captive conditions and humans, with the ultimate goal of modifying, generations after generations, selected traits, to produce, in most cases, more productive and efficient individuals (Bilio, 2008; Olesen et al., 2015; Houston et al., 2020).

## Question 2: What Is a Domesticated Fish?

For Balon (2004), animals become domesticated when they change form, function, color, and behaviour; often only partially resemble their wild ancestors; and survive poorly as feral forms if returned to the wild without human protection. For Bilio (2008), fish species are considered domesticated when they show first results of selective breeding or when no such evidence is found, after at least three successive cycles of reproduction (generations) under controlled conditions (the choice of three full cycles in captivity was an arbitrary criterion). Duarte et al. (2007) considered that fish are domesticated when breeding, caring, and feeding of organisms are controlled by humans. For Gjedrem et al. (2012), domesticated fish strains are the result of several generations of selection. Hence, rather than trying to define what a domesticated fish is, which could be *in fine* considered an arbitrary decision because it varies widely between authors and no clear threshold separates wild from domesticated animals (Teletchea, 2017), Teletchea and Fontaine (2014) coined a new concept (domestication levels) based on the degree of human control over the life cycle of farmed fish species. This domestication scale contains five levels (Table 1). According to this new classification, it was proposed that only fish species reaching at least the level 4 (full life cycle completed in captivity without wild inputs) could be considered domesticated. Yet, a domesticated fish is neither a definitive status as these animals continue evolving all the time (to cope with new captive conditions or because new traits are selected), nor a final end point of domestication because they can sometimes return to the wild, a process known as feralization (readaptation to the natural environment), which is one of the main issues of aquaculture globally (Lorenzen et al., 2012; Glover et al., 2017).

**Table 1.** Description of the domestication levels of fish species (modified from Teletchea, 2019b)

Level	Description	*n*_species_	*n*_families_	Three main families (*n*)
5	Selective breeding programs are applied focusing on specific goals	30	10	Cyprinidae (10), Salmonidae (8), Acipenseridae (5)
4	Full life cycle is controlled in captivity without the use of wild inputs	45	25	Cichlidae (6), Sparidae (5), Cyprinidae (4)
3	Full life cycle is controlled in captivity, yet wild inputs are still used	61	35	Sparidae (8), Cyprinidae (4), four families (3)
2	Only part of the life cycle is controlled in captivity due to key bottlenecks	75	39	Cyprinidae (9), Serranidae (5), Carangidae (4)
1	First trials of acclimatization to captive conditions	39	24	Cyprinidae (8), Sciaenidae (3), Siganidae (3)

*n*_species_, total number of species per level; *n*_families_, total number of families per level; *n*, number of species for the three main families.

## Question 3: How Many Fish Species Are Domesticated?

The number of fish species considered domesticated varies tremendously between authors from 2 for Balon (2004), 42 for Bilio (2008) to over 250 for Duarte et al. (2007). Yet, the number proposed by Balon (2004) is clearly too low because of his strict definition (see above). Conversely, the very high number documented by Duarte et al. (2007) simply reflects the growth of aquaculture globally (Hedgecock, 2012); farming a fish species does not necessarily imply that it has been domesticated (Bilio, 2008; Klinger et al., 2013; Teletchea and Fontaine, 2014). Among the 250 fish species listed by Duarte et al. (2007), which were established from the FAO database for the years 1950 to 2009, only one-third had reached the level 4 (*n* = 30) or level 5 (*n* =45) (Teletchea and Fontaine, 2014); which is much closer to Bilio’s estimations. Nearly a decade later, it is likely that new species have reached levels 4 and 5 (e.g., Teletchea, 2015b; Valladão et al., 2018) and probably 100 could be considered domesticated globally (see also FAO, 2019; Houston et al., 2020).

## Question 4: How Long Does It Take to Domesticate a Fish Species?

**Figure 1.**
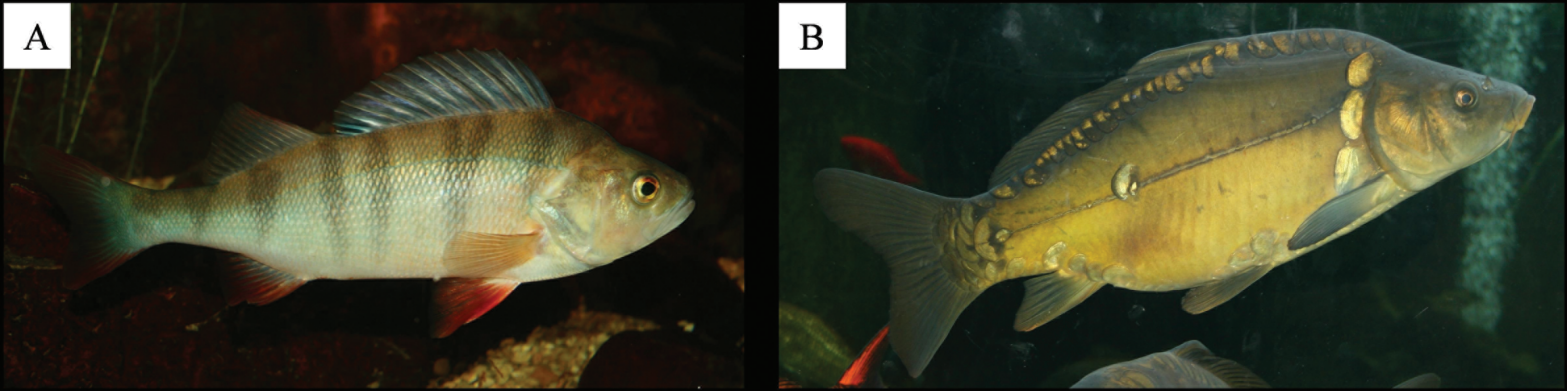
Examples of two freshwater fish species that have reached in 2009 the level 4: European perch *Perca fluviatilis* (A) and the level 5: common carp *Cyprinus carpio* (B). Pictures taken from www.storefish.fr (Teletchea and Teletchea, 2020).

Domesticating a fish species implies that the full life cycle is controlled in captivity without wild inputs (Table 1). The time required to reach this milestone varies tremendously between species, if ever attained (Bilio, 2008; Teletchea and Fontaine, 2014). Indeed, numerous farming trials of new species failed only after a few years mostly due to insufficient biological, ecological, and zootechnical knowledge (Teletchea and Fontaine, 2014). Key bottlenecks in closing the life cycle in captivity are (1) inadequate feeds, particularly for the first feeding of tiny larvae of numerous marine fish species, (2) poor gonadal development, and (3) lack of spawning (see also Bilio, 2008). Most often, we have no information about the domestication history of farmed species (Teletchea, 2019a); yet see for instance Gjedrem (2010, 2012) and Glover et al. (2017) for Atlantic salmon (*Salmo salar*) and Fontaine and Teletchea (2019) for Eurasian perch (*Perca fluviatilis*) (Figure 1A). In conclusion, domesticating a new fish species is a risky journey that may take years or even decades (Bilio, 2008; Teletchea and Fontaine, 2014; FAO, 2019).

## Question 5: Is Fish Domestication Going too Fast?

Once the full life cycle is controlled in captivity, there are no longer exchanges between farmed individuals and their wild congeners, and domestication can proceed toward the production of improved individuals (Table 1). For some domesticated species, several generations under selection have allowed improving specific traits very rapidly (Olesen et al., 2015; Nguyen, 2016; Teletchea, 2016b; Houston et al., 2020). Therefore, the time lag between the onset of domestication and selective breeding can be considerably short in aquaculture (less than a decade), with both occurring in tandem in many cases (Houston et al., 2020). However, it was found that without proper management, numerous breeding programs resulted also in a quick loss of genetic diversity because of inbreeding, possibly leading to a decline of productivity, a reduced population fitness, and an increased susceptibility to stress and disease (Olesen et al., 2015; Nguyen, 2016; Houston et al., 2020). Therefore, caution should be taken not to go too quickly when implementing breeding programs and adequately balance market (e.g., growth rate, fillet quality) and non-market values, such as ethics and welfare (Saraiva et al., 2019). Research has pushed the physiological limits of many fish species in growth, fertility, and size, as a consequence of (or resulting in) highly artificial conditions, possibly altering their welfare, which is one of the key issues of aquaculture today (Saraiva et al., 2019). It is also crucial to maintain sufficient genetic variability (e.g., establish a base population with ample genetic variability, keep a large effective population size, and introduce genetic variability from outside the breeding stock) of domesticated and selected fish to ensure that they are more robust and able to cope with various environmental changes (Olesen et al., 2015; Nguyen, 2016; Teletchea, 2016b). Supported by continuous advances in sequencing and bioinformatics, genomic tools appear now hugely valuable to inform sustainable genetic improvement and their affordability and accessibility mean that they can now be applied across the broad range of aquaculture species and at all stages of the domestication process to optimize selective breeding (Houston et al., 2020).

## Question 6: What Are the Most Domesticated Fish Species?

Thirty species belonging to 10 families have reached the level 5 (Table 1), including Acipenseridae (*n* = 5), Cichlidae (*n* =1), Cyprinidae (*n* = 10), Gadidae (*n* = 1), Ictaluridae (*n* = 1), Moronidae (*n* =1), Paralichthyidae (*n* =1), Salmonidae (*n* = 8), Scophthalmidae (*n* = 1), and Sparidae (*n* = 1) (Teletchea, 2019b). Among those 30 fish species, common carp (Figure 1B) and Nile tilapia are probably the most selected for the longest period of time globally (Bilio, 2008; Gjedrem et al., 2012; Nguyen, 2016; Teletchea, 2019a). In Europe, the most domesticated and selected species are common carp, rainbow trout (*Oncorynchus mykiss*), Atlantic salmon, gilthead sea bream (*Sparus aurata*), European seabass (*Dicentrarchus labrax*), and turbot (*Psetta maxima*) (Janssen et al., 2017).

## Question 7: Which Traits Were Selected?

Selective breeding programs in fish have historically focused on improving growth (Nguyen, 2016; Gjedrem and Rye, 2018). Genetic gain averages about 10% to 20% per generation for growth rate when this is the main, or only, selected trait (Gjedrem et al., 2012). In addition to growth, feed conversion efficiency, age at sexual maturity, improved resistance to bacterial and viral diseases, and a number of traits related to product quality (e.g., muscle lipid content, flesh color, tenderness, flavor) have been gradually included in various breeding programs, particularly for Atlantic salmon (Gjedrem, 2010, 2012). In a recent survey conducted among breeding companies of five species farmed in Europe, Janssen et al. (2017) found that growth performance was universally selected upon. Among the 27 breeding programmes, both morphology and disease resistance were included in 15, product quality in 13, processing yield in 12, and reproduction and feed efficiency in 7 (Janssen et al., 2017). In conclusion, the future seed market will most likely continue to request genetic material that is selected for growth rates as well as other traits (Olesen et al., 2015; see also Table 25 in FAO, 2019).

## Question 8: Is there a link between fish domestication level and production volume?

It is impossible today to definitively conclude whether domestication levels (Table 1) and production volumes are positively linked given the actual nature of data provided to the FAO by its members and associated nations (Klinger et al., 2013; Teletchea and Fontaine, 2013). Yet, Bilio (2008) highlighted that the percentage of domesticated species is increasing with the production level. The share of domesticated species is probably close to zero as long as the production per species remains below 100 tons and close to 100% for species reaching a production of 1 million tons (see Table 7 in Bilio, 2008). In other words, fully closing the life cycle in captivity seems positively related to a significant production: the top 15 most-produced species in 2009 all have reached level 4 or 5 (Teletchea and Fontaine, 2013). This includes species for which the onset of domestication is either centuries old, such as common carp or Nile tilapia, or a few decades old, such as Atlantic salmon (Teletchea, 2019b). In Europe, the proportion of aquaculture production that originates from selective breeding is very high, with a market share that exceeds 80% (Janssen et al., 2017). Atlantic salmon clearly appears as an outlier with close to 100% of the production that are now based on improved stocks (Gjedrem, 2010; Gjedrem et al., 2012). Yet, for most farmed species reaching level 4 or 5 does not necessarily imply that their total aquaculture production is based on this level (stocks of the same species can be at different domestication levels). Overall, only 10% of the global aquaculture production comes from genetically improved stocks (Gjedrem et al., 2012; Olesen et al., 2015).

## Question 9: Should We Stop Domesticating New Fish Species?

The boom of fish aquaculture has relied partly on the domestication of an increasing number of fish species, even though most domestication experiments stopped or failed to reach a significant volume and the global production is today heavily skewed toward the farming of a few species (FAO, 2019; Teletchea 2019b; Sicuro, 2021). The 20 most-produced species account for >84% of total production (FAO, 2019; Teletchea, 2019b). The main farmed species have been extensively introduced around the world (De Silva et al., 2009; Teletchea, 2019a). Seven of the eight most widely farmed fish species are more frequently reported by countries where they are non-native than by countries where that are native (FAO, 2019). For instance, common carp is farmed in 48 countries, among which 37 where it was introduced (FAO, 2019). Likewise, Nile tilapia is farmed in 45 countries (33 introduced) and rainbow trout in 45 countries (40 introduced) (FAO, 2019). The introduction of non-native species can affect biodiversity, directly or indirectly, and these impacts can be immediate or long term (De Silva et al., 2009). Therefore, reducing the dependence on non-native species, and thereby minimizing possible negative impacts on biodiversity, is increasingly perceived as an imperative for the sustainable development of aquaculture (De Silva et al., 2009). In this context, there are conflicting demands for further diversification versus the need to focus and improve the efficiency of production of existing farmed species (FAO, 2019). Bilio (2008) considered that it is no longer desirable to seek further diversification by subjecting yet more species to experimentation, but rather restrict our efforts to a few species and exploit intra-specific diversity potential, that is, the still largely unknown genetic diversity resources within truly domesticated species. Conversely, there is still huge potential for domesticating new fish species, particularly native ones, to develop a more diverse aquaculture sector likely to be more resilient to challenges of environmental change (Valladão et al., 2018; FAO, 2019; Fontaine and Teletchea, 2019). Such a strategy might also help to eliminate, or at least minimize, the adverse ecological and genetic impacts of either direct or indirect introduction of non-native species (De Silva et al., 2009). In recent years, the willingness to promote native species for aquaculture enterprise has resulted in significant changes in various countries, particularly in South America (Valladão et al., 2018). For example, the production of pacu *Piaractus mesopotamicus* has increased significantly in recent years, exceeding the production of the non-native rainbow trout in 2012 in Argentina (Valladão et al., 2018). The contribution of native species to global aquaculture will perhaps increase, resulting in a more diversified and even production than today (Teletchea, 2019b). In conclusion, it is likely that both intra- and interspecific diversification will be pursued at least in the coming decade, that is to continue improving already domesticated and selected species and to farm new fish species (FAO, 2019; Teletchea, 2019b).

## Question 10: Do We Already Need a Sixth Level of Domestication?

Given the tremendous progresses in fish domestication, it might be timely to propose a sixth level of domestication for the species for which selection has resulted in strains. According to the FAO (2019), a strain is a “farmed type of aquatic species having homogeneous appearance (phenotype), homogeneous behaviour and/or other characteristics that distinguish it from other organisms of the same species and that can be maintained by propagation.” Some strains (notably for common carp and rainbow trout) are already officially registered in a limited number of countries (Bilio, 2008), but there are still very few distinct, stable, and reproducible strains in aquaculture (Bilio, 2008; FAO, 2019). One very well-known example is the genetically improved farmed tilapia (**GIFT**) strain developed in the early 1990s from a base population including wild and farmed strains from eight African and Asian countries (Gjedrem, 2012; Nguyen, 2016; Houston et al., 2020). The GIFT strain is now farmed in 16 countries across Asia, Africa, and Latin America and grows 85% faster than the base population (Houston et al., 2020). Similarly, the Atlantic salmon is certainly the fish for which the domestication history is best known (Teletchea, 2019b) and was the first species to be subject to a systematic family-based breeding program (Gjedrem, 2010, 2012). Currently, over 12 generations have been consecutively bred in captivity for the oldest breeding programs in Norway and multiple strains were established in several countries (Glover et al., 2017). Nevertheless, it might still be too early to propose a sixth level of domestication for only a few strains in a handful of species; this situation might change quickly, and many recognizable strains could be soon officially recognized and registered as observed for the thousands breeds in farmed land mammals and birds (FAO, 2019).

## Conclusions

Domestication is a long and endless process that allows adapting fish to both captive conditions and humans. This process started only a few decades (or even years) ago for most farmed species, and therefore probably less than one-third could be considered domesticated. Several traits, among which growth, were modified during domestication. New breeding programs will need to balance market and non-market values while maintaining a sufficient genetic variability to ensure that fish are productive as well as robust enough to cope with various environmental changes. The sustainable future of aquaculture will rely first on the continuous improvement of already domesticated fish species and second on our willingness and capacity to diversify the number of farmed, preferably native, species to promote a more diversified and even aquaculture production.

## About the Authors

Fabrice Teletchea studied marine biology and then systematics in France. Since 2010, he is associate professor at the University of Lorraine in Nancy, France. He first worked on fish taxonomy and then moved to the study of fish domestication in aquaculture. He developed a comparative framework of the reproductive strategies of European freshwater fish species in order to better understand the different trade-offs observed at the early-life stages (www.storefish.org), to help domesticating them more efficiently. Besides his research and teaching activities, he is in charge of a professional bachelor entitled “Inland Aquaculture and Aquariology” for nearly 10 years at the IUT Nancy-Brabois.
